# Hydrodynamic role of longitudinal dorsal ridges in a leatherback turtle swimming

**DOI:** 10.1038/srep34283

**Published:** 2016-10-03

**Authors:** Kyeongtae Bang, Jooha Kim, Sang-Im Lee, Haecheon Choi

**Affiliations:** 1 Department of Mechanical & Aerospace Engineering, Seoul National University, Seoul, Korea; 2 School of Mechanical and Nuclear Engineering, Ulsan National Institute of Science and Technology, Ulsan, Korea; 3 Institute of Advanced Machines and Design, Seoul National University, Seoul, Korea; 4 Laboratory of Behavioral Ecology and Evolution, School of Biological Sciences, Seoul National University, Seoul, Korea

## Abstract

Leatherback sea turtles (*Dermochelys coriacea*) are known to have a superior diving ability and be highly adapted to pelagic swimming. They have five longitudinal ridges on their carapace. Although it was conjectured that these ridges might be an adaptation for flow control, no rigorous study has been performed to understand their hydrodynamic roles. Here we show that these ridges are slightly misaligned to the streamlines around the body to generate streamwise vortices, and suppress or delay flow separation on the carapace, resulting in enhanced hydrodynamic performances during different modes of swimming. Our results suggest that shapes of some morphological features of living creatures, like the longitudinal ridges of the leatherback turtles, need not be streamlined for excellent hydro- or aerodynamic performances, contrary to our common physical intuition.

Leatherback sea turtles (*Dermochelys coriacea*), the largest and the deepest diver among marine turtles, are known to have superior diving ability^1^,^2^,^3^. They are also known for long-distance migration and considered to be highly adapted to pelagic swimming^2^,^4^,^5^,^6^,^7^. On the body of leatherback turtles, there are a few remarkable morphological features such as soft carapace, big flippers, and longitudinal carapace ridges that distinguish them from other marine turtles^8^. Among these, five longitudinal ridges on their carapace are a notable feature. Some conjectured that these ridges represent an evolutionary adaptation for keeping the flow around the body laminar^9^,^10^. Some of nature’s morphological features have been shown to provide better aero- and hydrodynamic performances. For example, dorsal and ventral keels of a boxfish generate streamwise vortices, and these vortices are considered to increase the hydrodynamic stability^11^,^12^,^13^; tubercles on the leading edge of a humpback whale’s flipper increase the lift by generating streamwise vortices and delaying separation^14^,^15^,^16^; an alula on the leading edge of a bird’s wing produces a streamwise vortex and increases the lift^17^; a serrated leading edge of an owl’s feather also produces streamwise vortices to fly silently^18^,^19^; spade-like protrusions on the trailing edge of a dragonfly wing provide an idea for reducing drag on an airfoil with a gurney flap^20^. All of these morphological features are located on the leading edges of the wing and flipper, on the frontal part of the body, and on the trailing edge of the wing. Unlike these morphological features, the longitudinal ridges of a leatherback sea turtle are located along the entire body. In this respect, the hydrodynamic roles of ridges in leatherback turtle’s swimming should be interesting to investigate. However, no study has been made for their hydrodynamic roles yet. Therefore, in the present study, we investigate their roles in the conditions that represent the swimming modes of hatchling and adult leatherback turtles.

The diving patterns of leatherback turtles are divided into the V-shaped diving, U-shaped diving, and sub-surface swimming according to the shapes of the diving profile^3^,^5^,^21^,^22^ (see, for example, Supplementary Fig. S1). The V-shaped diving, used for foraging and transit, is a typical diving pattern of adult leatherback turtles^3^,^5^,^21^,^22^. Breath-hold divers such as leatherback turtles have no buoyancy-control organ and thus experience negative buoyancy in deep water due to the compression of pulmonary air by water pressure^5^,^23^. This negative buoyancy enables diving, so their descending swim can be largely energy-efficient^5^,^23^. In contrast, they have to actively swim up at high pitch angles (or high angles of attack) during the ascending period to overcome the negative buoyancy^5^. Therefore, the hydrodynamic performance in ascending swimming conditions at positive angles of attack can be energetically important for the leatherback turtles.

On the other hand, hatchling or juvenile leatherback turtles cannot dive as deep as adults since they cannot hold their breath for a long time due to the low tissue volume for oxygen storage and high mass-specific metabolic rates^24^,^25^. Therefore, hatchlings swim mainly in shallow water^25^. Their swimming patterns are divided into a routine swimming (slow) near the water surface and a vigorous swimming (fast and large energy consuming) underwater^26^. The vigorous swimming is important for hatchlings as it provides a means to overcome positive buoyancy and escape from predators^26^,^27^. Therefore, the hydrodynamic characteristics during the vigorous swimming (swimming at large negative angles of attack) should also be considered to fully understand the roles of the longitudinal ridges.

In the present study, we constructed carapace models of a leatherback turtle with and without the longitudinal ridges, and conducted force and velocity measurements to investigate the hydrodynamic roles of the ridges in different modes of swimming of the leatherback turtles. A carapace model for wind tunnel experiments was constructed based on a geometric information of a stuffed leatherback turtle at National Science Museum, Daejeon, Korea. To understand the effect of the ridges on the drag and lift forces and flow near the body, a carapace model without the ridges but with same frontal (*A*_*f*_) and planform (*A*_*p*_) areas was also constructed (Fig. 1) through mathematical approximation of geometries (details are given in Methods (Carapace models) and Supplementary Fig. S2).

We conducted a series of wind tunnel tests to measure the drag and lift forces on the models with and without the ridges (see Methods (Force measurements) and Supplementary Fig. S4 for detailed setup). The Reynolds numbers considered were *Re* = 0.2–1.2 × 10^6^ (based on the body length *l* of each model) with varying the angle of attack from *α* = −22° to 22°. These *Re* and *α* ranges include swimming conditions of hatchling and adult leatherback turtles (Supplementary Fig. S1c). We also considered non-zero yaw angles for *α*  =  −22° and 18°, and measured the side forces (Supplementary Fig. S4).

Figure 2a shows the variations of the drag and lift coefficients (*C*_*D*_ and *C*_*L*_, respectively; see Methods (Force measurements) for their definitions), and lift-to-drag ratio (*L*/*D*) depending on the angles of attack (*α*) and the Reynolds numbers (*Re*). This figure demonstrates two noticeable hydrodynamic roles of the ridges. First, the ridges significantly reduce the drag and also reduce the negative lift at negative to near zero angles of attack. Especially, at low Reynolds numbers, the drag is reduced by up to 32% at *α* = −18° (Fig. 2b). In the vigorous swimming, hatchling leatherback turtles adopt a head down/tail up posture at which the attack angle of the body is about −22° 26. At this large negative angles of attack, the drag is large and the lift is negative (as shown in Fig. 2a). Our results suggest that the ridges reduce this high drag force generated during the vigorous swimming of hatchlings.

Let us estimate the importance of these drag and negative-lift reductions for a hatchling leatherback turtle during the vigorous swimming. At *Re = U*_o_ *l*/ν = 2 × 10^5^, the swimming speed (*U*_o_) and body length (*l*) of a hatchling leatherback turtle are obtained to be *U*_o_ = 0.783 m/s (≈3.3 *l* s^−1^)^26^ and *l* = 23.8 cm (the shortest body length of a hatchling leatherback turtle is known to be 9 cm^26^), where the kinematic viscosity of sea water is ν = 0.932 × 10^−6^ m^2^/s^28^ and the density of sea water is *ρ* = 1030 kg m^−3^. Its body mass (*m*) is 1.89 kg^29^, body volume (*V*_o_) is 1.94 × 10^−3^ m^3^ 5,30, and planform area is *A*_*p*_ = 0.0260 m^2^. Since we do not find any information of the measured thrust of a leatherback turtle during the vigorous swimming, we estimate it based on the thrust of a vigorously swimming green sea turtle (*Chelonia mydas*) assuming that the vigorous swimming is a typical swimming pattern of sea turtle hatchlings^31^. According to Davenport *et al*.^32^, the body length of a green sea turtle hatchling is 11 cm, and its thrust during the vigorous swimming is 0.61 N (average thrust over 5 s). Then, the thrust of a vigorously swimming leatherback turtle is estimated to be about 2.86 N, assuming that the thrust is proportional to the square of the body length^33^. The drag coefficients at *Re* = 2 × 10^5^ and *α* = −22° are 0.127 and 0.15 with and without the ridges, respectively. Thus, the amount of drag reduction by the longitudinal ridges is about 0.19 N, which is 6.6% of the thrust force during the vigorous swimming. Therefore, the amount of drag reduction by the ridges is quite remarkable during the vigorous swimming. On the other hand, the lift coefficients during the vigorous swimming (*Re* = 2 × 10^5^ and *α* = −22°) are −0.291 and −0.331 with and without the ridges, respectively. Then the lift forces during the vigorous swimming with and without the ridges are −2.39 N and −2.72 N, respectively. The buoyancy force by sea water on a hatchling leatherback turtle is *B* = *ρV*_o_*g* = 19.6 N. Therefore, the net forces on the hatchling turtles with and without the ridges are *B*–*mg* + *L* = −1.33 N and −1.66 N, respectively. This analysis shows that the longitudinal ridges reduce negative net vertical force by about 20% but still maintain negative value of the vertical force on the body of a hatchling leatherback turtle. Unnecessarily large net vertical force can make it difficult for hatchlings to control the swimming direction. Therefore, the reduced negative net vertical force can be beneficial for vigorously swimming hatchlings.

On the other hand, vigorously swimming hatchlings show large periodic flipper motions, resulting in non-uniform swimming speed^26^. The range of swimming speed is 0.738–1.714 m/s (3.1–7.2 *l* s^−1^)^26^ for the body length (*l*) of 23.8 cm, whose corresponding Reynolds numbers are *Re* = 1.9–4.4 × 10^5^. At these Reynolds numbers and *α* = −22°, the longitudinal ridges reduce the drag and increase the lift (Fig. 2b), indicating that the hydrodynamic performance of the ridges is still maintained during the flipper motion.

Second, at positive high angles of attack, the ridges increase both the drag and lift forces, and the lift-to-drag ratio. Especially, at relatively high Reynolds numbers, the lift coefficient and lift-to-drag ratio increase by up to 16% and 7%, respectively, whereas the drag coefficient increases by 5.6% (Fig. 2b). During active ascending swimming, the angle between the swimming and horizontal directions (*θ* in Supplementary Fig. S1) is 20^o^ –30^o^^5^. So, the changes in the forces opposite and perpendicular to the gravity direction (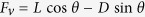 and 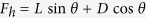, respectively), and their ratio due to the ridges are also similar to those of *L*, *D* and *L*/*D*, respectively: e.g., for *θ* = 30^o^, *F*_*v*_ and *F*_*v*_/*F*_*h*_ increase by 18% and 5%, respectively. High Reynolds numbers are characteristic of the swimming mode of active ascent by adult leatherback turtles in the V-shaped diving^5^. Our results suggest that the hydrodynamic performance can be enhanced by the ridges in the V-shaped diving where lift generation is required due to negative buoyancy during most of their ascent. Therefore, the longitudinal ridges on the carapace of leatherback turtles provide superior hydrodynamic performance by reducing the drag in hatchlings’ swimming and generating additional lift in adults’ swimming.

We conducted velocity measurements using DPIV to investigate the modifications of flow structures by the longitudinal ridges. Measurements were performed at two conditions, *α* = −22° and *Re* = 2 × 10^5^, and *α* = 18° and *Re* = 5 × 10^5^, that are characteristic of the vigorous swimming of hatchlings and the active ascending swimming of adults, respectively (see Methods (Flow-field measurements) and Supplementary Fig. S5 for the experimental setup).

Figure 3 shows the flow fields at *α* = −22° and *Re* = 2 × 10^5^ (vigorous swimming of hatchlings) at which the ridges reduced the drag by 15.5% and increased the lift by 12% (see Fig. 2b). As shown in Fig. 3a, the flow separates at rear part of the body (separation starts from *x*/*l* = −0.21) in the absence of the ridges. With the ridges, flow separation is significantly delayed (there is no separation at *z = z*_*1*_ and separation starts from *x*/*l* = −0.06, −0.18, and −0.12 at *z = z*_*2*_, *z*_*3*_, and *z*_*4*_, respectively), which is the main reason of drag decrease and lift increase by the ridges. To understand the mechanism of separation delay by the ridges, the contours of instantaneous vorticity and velocity vectors on four cross-flow (*y*-*z*) planes are drawn in Fig. 3b. In the absence of the ridges, the shear layer instability occurs after flow separation (see the flow at *x = x*_3_) and strong streamwise vortices are generated at a downstream location (*x = x*_*4*_). On the other hand, with the ridges, flow locally separates across the first off-center ridge at *x = x*_1_ because this ridge is not aligned to local streamlines. Then, a shear layer evolves, and streamwise vortices are generated at *x = x*_2_ and get stronger further downstream. These strong streamwise vortices bring momentum to the flow near the surface, and enable the flow to resist the adverse pressure gradient and to delay the separation. Although the local separation at *x = x*_1_ increases the drag, the drag reduction from the main separation delay is much larger than this drag increase, resulting in a significant decrease of total drag. Therefore, the mechanism responsible for main separation delay by the ridges is the generation of streamwise vortices through a local separation by the ridges.

Figure 4 shows the flow fields at *α* = 18° and *Re* = 5 × 10^5^ (active ascending swimming of adults) at which both the drag and lift are increased by 5.6% and 11%, respectively, thereby resulting in the increase of the lift-to-drag ratio by 5% by the ridges. From the surface-oil visualization (Fig. 4a), separation and reattachment lines denoted as solid and dashed red lines, respectively, are formed at the front part of the body in the absence of the ridges. On the other hand, with the ridges, separation occurs only locally near the centerline of the front part. At the rear part of the body, however, it was almost impossible to identify flow structures from the surface-oil visualization because the oil moved downward due to the steeply inclined rear surface. Therefore, separation lines are obtained from velocity measurements above the rear surface. Figure 4b shows that the separation line on the rear surface is broadened with the ridges. This result indicates that the ridges suppress the formation of separation bubble existing on the front surface but enhance the separation on the rear surface. Without the ridges, the separation bubble observed on the front surface is similar to that on a low Reynolds number airfoil causing its performance deterioration^34^,^35^,^36^. Thus, with the ridges, the hydrodynamic performance is increased owing to the reduced separation bubble on the front surface. To explain the suppression of front-body separation by the ridges, contours of the instantaneous streamwise vorticity on four *y*-*z* planes for both models are shown in Fig. 4c. In this figure, the spanwise domain is in between the first and second off-center ridges, where weak separation exists without the ridges and no separation occurs with the ridges (Fig. 4a). Without the ridges, flow separation is so weak that there is no strong shear layer evolution. On the other hand, the second off-center ridge which is misaligned with local streamlines produces local flow separation in the spanwise direction at *x*_*1*_. Then, as the fluid flows downstream, strong streamwise vortices are produced at *x*_*2*_–*x*_*4*_. These streamwise vortices enable the flow to resist the adverse pressure gradient by supplying momentum to the flow near the surface, which in turn removes the separation on the front surface in between the first and second off-center ridges. Although a part of the separation bubble is suppressed by the ridges, the drag force on the model with the ridges is greater by 5.6% as compared to that without the ridges. This is because the flow separates earlier on the rear surface with the ridges than without the ridges (Fig. 4b). The early separation at *z*/*l* = 0.04 is caused by the ridge itself. Therefore, this broadened separated region on the rear surface with the ridges leads to the increase in the form drag because this part of surface is nearly vertical at this high angle of attack. On the other hand, as discussed above, the ridges suppress the separation bubble at the front surface where the surface is nearly horizontal, and thereby enhance the lift force by 11%. This increase in the lift contributes to the increase in the lift-to-drag ratio, although the drag is increased.

Since the separation bubble exists on the front surface of the model for the active ascending swimming, the presence of turtle’s head may affect the flow above the front surface. According to previous observations, the angle of the head from the body of a leatherback turtle does not noticeably change during swimming^26^,^37^. Therefore, we constructed additional carapace models including the head part by scanning the head of a leatherback turtle (Supplementary Fig. S3) and measured the drag and lift forces. In the case of high attack angle and high *Re* condition that represents the active ascending swimming of adults, the ridges enhance both the lift (9.3%) and lift-to-drag ratio (5.2%) even in the presence of the head (see Supplementary Fig. S7a). On the other hand, in the case of negative attack angle and low *Re* condition that represents the vigorous swimming of hatchlings, the ridges significantly reduce the drag (up to 22%) and increase the lift (up to 23.3%) (Supplementary Fig. S7b). These results indicate that the presence of the head part does not change the hydrodynamic roles of the ridges for both the vigorous and active ascending swimming.

In the presence of ocean current or during turning motion of a turtle, the swimming direction does not coincide with the freestream direction. To examine this effect, we measure the drag, lift and side forces by varying the yaw angle from *γ* = 0° to 30° (Supplementary Fig. S4) for both the vigorous and active ascending swimming, and present their variations in Fig. 5. At *α* = −22^o^ and *Re* = 2 × 10^5^ (vigorous swimming of hatchlings; Fig. 5a), the ridges reduce the drag and increase the lift at *γ* = 0° to 30°. The side force rapidly increases with increasing yaw angle. The ridges increase the side force more at a large yaw angle of 30^o^, although they do not change it much at low yaw angles. At *α* = 18^o^ and *Re* = 5 × 10^5^ (active ascending swimming of adults; Fig. 5b), the ridges enhance the lift and lift-to-drag ratio. On the other hand, the side force shows very different behaviors: i.e., without the ridges, the side force becomes negative at low yaw angles and then positive at a large yaw angle of 30°, whereas it continuously increases with increasing yaw angle with the ridges. These results suggest that, unless the yaw angle is very large, the hydrodynamic roles of the longitudinal ridges in terms of the lift, drag, and their ratio are still similar to those of zero yaw angle.

Leatherback turtles have a vaulted carapace, whereas other hard-shelled sea turtles have relatively flat carapaces. Typically, the flow over a vaulted surface may experience stronger adverse pressure gradient followed by flow separation^38^. Therefore, leatherback turtles, unlike other sea turtles, may have a higher probability of massive flow separation on their carapace, so any device that aids separation delay should be more useful in swimming than other turtles having rather flat carapaces. Also, in contrast to the V-shaped diving of leatherback turtles, other sea turtles (*Chelonia mydas, Caretta caretta*) are generally known for swimming at a location where they achieve a neutral buoyancy, and thus they may not experience a negative buoyancy during their swimming^31^,^33^. Therefore, the lift enhancement by the ridges in V-shaped diving may not be necessary in other sea turtles’ swimming.

The present results are the first experimental evidence about the hydrodynamic roles of the longitudinal ridges in leatherback turtle swimming. The ridges functioned differently depending on the flow conditions: (1) they significantly reduced the drag forces at negative angles of attack and relatively low Reynolds numbers that represents a vigorous swimming performed by hatchlings; (2) at positive angles of attack and relatively high Reynolds numbers, corresponding to an active ascending swimming of adults in V-shaped diving, the ridges enhanced both the lift and lift-to-drag ratio. Our DPIV results provided explanations on how the ridges enhanced the hydrodynamic performances for these two swimming conditions. The longitudinal ridges that are misaligned with local streamlines generated local flow separation, which in turn induced a shear layer instability and produced streamwise vortices (therefore, unlike the conjectures by Deraniyagala^9^ and Hendrickson^10^, the role of ridges is not to keep the flow over the carapace laminar). These streamwise vortices delayed or suppressed flow separation, resulting in both drag reduction for the vigorous swimming of hatchlings and lift enhancement for the active ascending swimming by adults. Other morphological features, such as dorsal and ventral keels of a boxfish^11^,^12^,^13^, tubercles on the leading edge of a humpback whale’s flipper^14^,^15^,^16^, an alula on the leading edge of a magpie’s feather^17^, and a serrated leading edge of an owl’s feather^18^,^19^, have shown aero- and hydrodynamic roles similar to that of the longitudinal ridges, in that they generate streamwise vortices and increase the aero- and hydrodynamic performances. However, there also exist notable differences between those cases and the present one. That is, those morphological features exist only at the frontal parts of the body, flipper and wing, and thus most of them work only at limited situations like at high angles of attack for lift enhancement. On the other hand, the longitudinal ridges exist along the entire body and function differently depending on the swimming conditions: i.e., the ridges reduce the drag at negative angles of attack by controlling the flow at the rear part of the body, and increase the lift at positive angles of attack by controlling the flow at the frontal part of the body. Our study therefore reveals a nature’s solution, i.e., the longitudinal ridges that are slightly misaligned to local streamlines, for flow control on a teardrop-shaped body at negative to positive angles of attack. These ridges are also contrary to a general intuition that a streamlined body shape is advantageous in decreasing the drag. Our results suggest that the hydrodynamic performance can be optimized even with the features that are not streamlined. We expect that the longitudinal surface protrusions slightly misaligned with local streamlines may provide an innovative design concept for vehicles with better hydro- and aerodynamic performances.

## Methods

### Carapace models

We constructed a carapace model based on three-dimensional surface data obtained by scanning a stuffed leatherback turtle (carapace length of 1.2 m, adult) at National Science Museum, Daejeon, Korea (Supplementary Fig. S2). According to previous observations, leatherback turtles have a relatively narrow range of motion in their neck and thus their head does not noticeably move relative to the carapace during swimming^26^,^37^. As for fore flippers, leatherback turtles show only synchronous flapping during linear progression^39^,^40^. For this reason, we assumed that the motions of head and flippers do not noticeably affect the flow near the carapace, and thus we eliminated the head and limb parts in the process of constructing a carapace model.

The carapace model was constructed such that its shape was similar to the body shape of the stuffed leatherback turtle. First, we measured the profile of the longitudinal ridge at the center (*z* = 0) of the stuffed leatherback turtle, and obtained a smooth profile of the ridge by applying a least square method based on 8^th^-order polynomials,


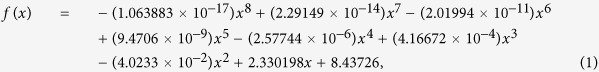


where 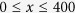. The spanwise edge of the carapace was located at 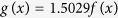 (see Supplementary Fig. S2).

The upper surface of the carapace model was divided by three parts in the streamwise direction: 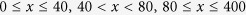, respectively. For 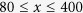, the carapace has three curved surfaces in the spanwise direction because of the ridges (Supplementary Fig. S2b), and thus each surface was modeled using a least square method based on 5^th^-order polynomials:


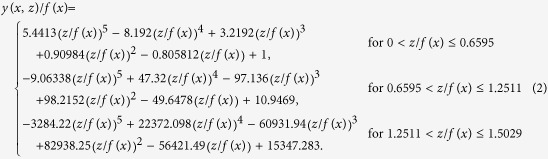


For 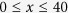, a smooth surface without the ridges was constructed using an ellipse following the body shape of the stuffed leatherback turtle:


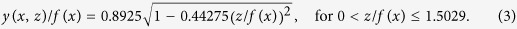


For 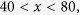 a surface shape of the carapace from smooth 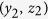 to curved 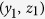 surface was constructed using a weighting factor between these two surfaces:


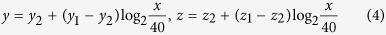


Here, 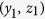 was first obtained for 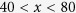 using Eq. (2), and then 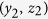 was obtained from 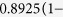

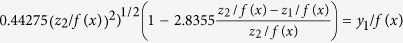
 and 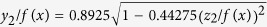.

The lower surface of the stuffed leatherback turtle was modeled using an ellipse:


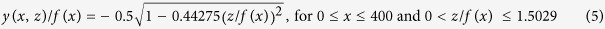


**Figure 4:**
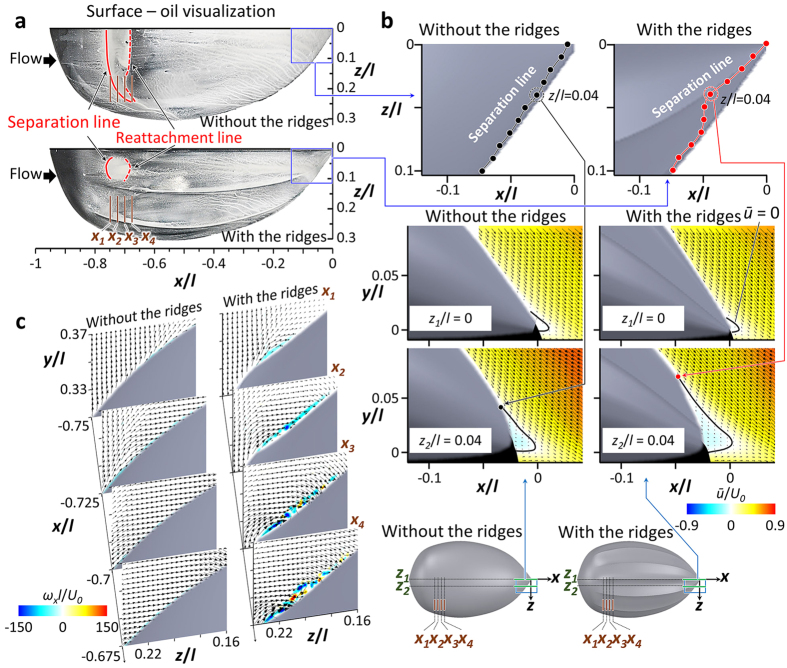
Results of flow-field measurements at *α* = 18° and *Re* = 5 × 10^5^ that represents the active ascending swimming of adults (*γ* = 0^o^). (**a**) Surface-oil visualization on the models without (upper panel) and with (lower panel) the ridges. Red solid and dashed lines denote the locations of flow separation and reattachment on the front surface of the model, respectively. (**b**) Contours of time-averaged streamwise velocity and velocity vectors at two spanwise locations on the rear part of the models without (left panel) and with (right panel) the ridges. Solid black lines denote the locations where the time-averaged streamwise velocity is zero. Separation lines in (**b**) (upper panel) are drawn from the velocity fields measured at eleven spanwise locations and detailed information is given in Supplementary Fig. S6. (**c)** Contours of the instantaneous streamwise vorticity and velocity vectors at four streamwise locations on the models without (left panel) and with (right panel) the ridges.

As we show in this paper, we observe that a separation bubble exists in the front part of the carapace surface at the swimming mode of active ascent (Fig. 4). Therefore, we made another set of carapace models including the head part by scanning a leatherback turtle’s head (Supplementary Fig. S3), to see how the head affects the flow over the carapace.

The length of the carapace model (*l*) was 400 mm, which was 1/3 scale of the carapace of the stuffed leatherback turtle. We also constructed a carapace model without the ridges (i.e. smooth surface model) for comparison by keeping the frontal (*A*_*f*_) and planform (*A*_*p*_) areas the same as those of the model with the ridges. In our analysis, we also assumed that hatchlings and adults are geometrically similar^41^. Thus, we used same carapace models to investigate the hydrodynamic roles of the ridges for different swimming modes of both hatchlings and adults, although the present carapace models were constructed from the geometric information of an adult leatherback turtle.

### Force measurements

The lift (*L*) and drag (*D*) forces on the carapace models with and without the ridges were measured in a wind tunnel. Various Reynolds numbers (*Re*) and angles of attack (*α*) were chosen considering the swimming conditions of both hatchling and adult leatherback turtles (Supplementary Fig. S1). The angles of attack (angle between the swimming direction and the body alignment) considered were −22°−22°, and the Reynolds numbers (*Re = U*_*0*_*l/ν*) were 0.2–1.2 × 10^6^, where *U*_*0*_ is the free-stream velocity, *l* is the model length, and *ν* is the kinematic viscosity of air. The lift (*L*) and drag (*D*) forces on both models were measured simultaneously with two load cells (A&D LCB03–015 M for the lift force and A&D LCB03-003 M for the drag force) (Supplementary Fig. S4). Resolutions of these two load cells were 0.015 N and 0.003 N with maximum capacities of 150 N and 30 N, respectively. The signals from these load cells were digitized by an A/D converter (PXI-6259, National Instruments Co.) and sampled for 60 s at a rate of 10 kHz to obtain the mean value. The repeatability errors of force measurements were within 2%. The lift (*C*_*L*_) and drag (*C*_*D*_) coefficients were defined as *C*_*L*_* = L*/(0.5*ρU*_*0*_^*2*^*A*_*p*_), *C*_*D*_* = D*/(0.5*ρU*_*0*_^*2*^*A*_*p*_), respectively, where *ρ* is the air density, and *A*_*p*_ is the planform area of the model at *α* = 0°. The carapace model was fixed using a strut which was directly mounted to the load cells (Supplementary Fig. S4). The wind tunnel used was a closed-type wind tunnel (Göttingen type) whose test section size was 0.9 × 0.9 m^2^. The blockage ratios due to the model were about 2.6% and 4.1% for *α* = 0° and *α* = 22°, respectively. To minimize the disturbance from the strut, its cross-section was designed to be an ellipse with a ratio of major to minor axis of 2. The height of the strut was adjusted to locate the carapace model at the center of the wind tunnel. The force on the isolated strut was measured separately and used for correction from those measured with the model.

In the presence of ocean current or during turning motion of a turtle, the swimming direction does not coincide with the freestream direction. This effect was examined by considering the yaw angle (*γ*) as shown in Supplementary Fig. S4. For non-zero *γ’*s, we measured the side forces in addition to the drag and lift forces.

### Flow-field measurements

We used a digital particle image velocimetry (DPIV) to obtain the velocity and vorticity fields around the carapace models with and without the ridges. The measurements were performed for two cases, *α* = −22° and *Re* = 2 × 10^5^, and *α* = 18° and *Re* = 5 × 10^5^, which represent the vigorous swimming of hatchlings and the active ascending swimming of adults, respectively. The same wind tunnel used for force measurements was used. The schematic diagram for DPIV is shown in Supplementary Fig. S5. The DPIV system consisted of an Nd:Yag laser (Dual Power 135-15, Litron), a laser optics (Short Mirror Arm, Dantec Dynamics), a pulse generator (IDT USB Timing Hub XS-TH, Integrated Design Tools), a fog generator (F2010, Safex), and a CCD camera mounted with an optical lens (APO MACRO 180 mm F2.8, SIGMA). A thickness of a laser sheet generated by the laser optics was about 2 mm. The fog generator produced liquid droplets which were spread inside the wind tunnel and their mean diameter was about 1μm. The velocity measurements were performed on various planes parallel to the *x*-*y* and *y*-*z* planes, respectively, where *x*, *y*, and *z* denote the streamwise, vertical, and spanwise directions, respectively, and the origin was located at the center of rear edge of the model. To obtain the velocity field from recorded images, an iterative cross-correlation analysis was performed with an initial window size of 64 × 64 pixels and a final interrogation window size of 16 × 16 pixels. The interrogation window was overlapped by 50%, leading to spatial resolutions of about 0.15 mm (3.75 × 10^−4^ *l*) on *x*-*y* planes and 0.23 mm (5.75 × 10^−4^ *l*) on *y*-*z* planes, where *l* is the model length. To obtain the time-averaged flow field, 2,000 pairs of images were collected and processed.

## Additional Information

**How to cite this article**: Bang, K. *et al*. Hydrodynamic role of longitudinal dorsal ridges in a leatherback turtle swimming. *Sci. Rep*. **6**, 34283; doi: 10.1038/srep34283 (2016).

## Supplementary Material

Supplementary Information

## Figures and Tables

**Figure 1:**
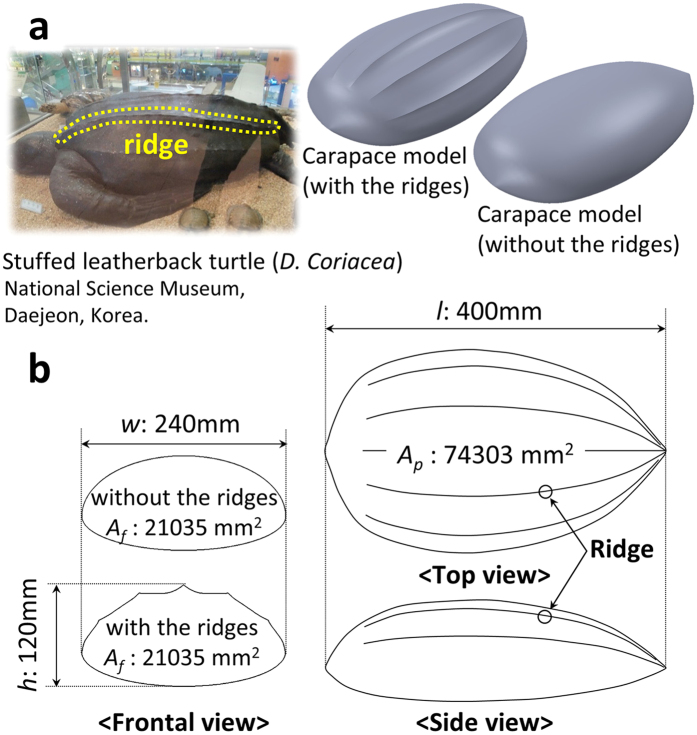
Carapace model specification. **(a)** Stuffed leatherback turtle used for 3D scanning, and perspective views of the carapace models with (left) and without (right) the ridges. **(b)** Characteristic lengths and areas of the carapace models.

**Figure 2:**
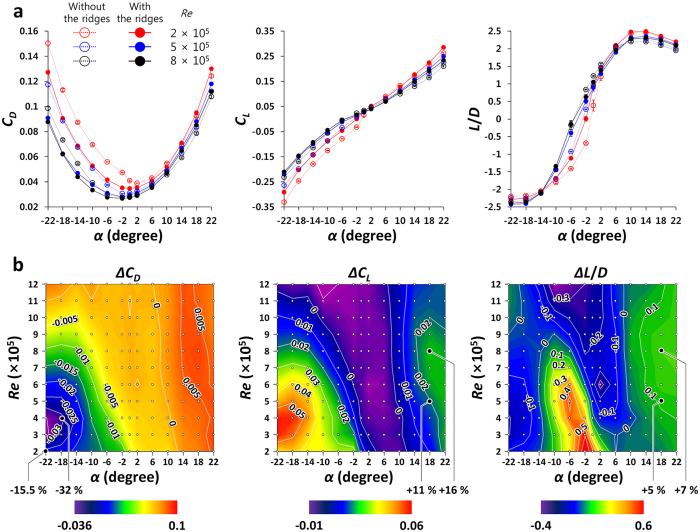
Results of force measurements (*γ* = 0^o^). **(a)** Variations of the drag (*C*_*D*_) and lift (*C*_*L*_) coefficients, and lift-to-drag ratio (*L*/*D*) on the models with (solid symbols) and without (open symbols) the ridges depending on the angles of attack (*α*) and Reynolds numbers (*Re*). Here, results for three representative Reynolds numbers are shown, and error bars denote the repeatability error of force measurements. **(b)** Variations of *C*_*D*_, *C*_*L*_, and *L*/*D* by the ridges. Here, *ΔC*_*D*_* = C*_*D with the ridges*_−*C*_*D without the ridges*_, and *ΔC*_*L*_ and *ΔL/D* are similarly defined. The white dots in this figure represent the points where the experiments were conducted.

**Figure 3:**
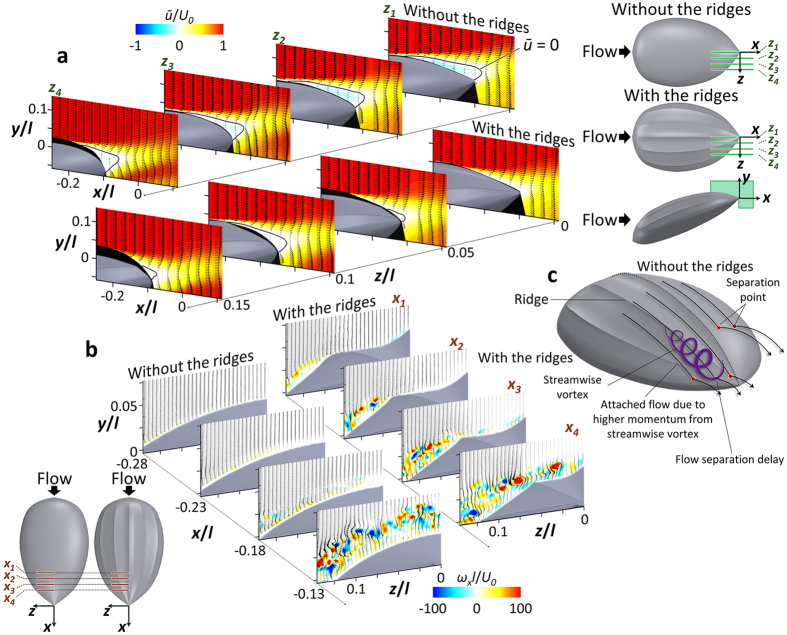
Results of flow-field measurements at *α* = −22° and *Re* = 2 × 10^5^ that represents the vigorous swimming of the hatchlings (*γ* = 0^o^). (**a**) Contours of the time-averaged streamwise velocity (*ū*) and velocity vectors at four spanwise locations (*z*_*1*_–*z*_*4*_) for the cases without (upper panel) and with (lower panel) the ridges. Solid black lines in this figure indicate the locations of *ū* = 0. Black-colored areas denote the regions where the velocity was not measured due to the surface reflection and the shadow of the model. (**b**) Contours of instantaneous streamwise vorticity and velocity vectors at four streamwise locations (*x*_*1*_–*x*_*4*_) for the cases without (left panel) and with (right panel) the ridges. (**c**) Mechanism of streamwise vortex generation and separation delay by the ridges.

**Figure 5:**
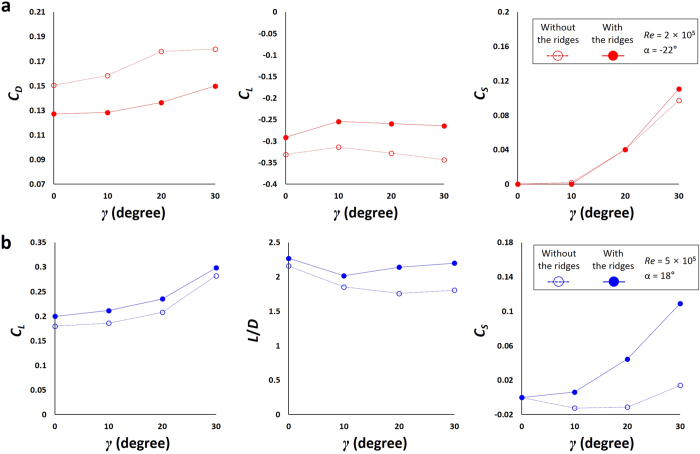
Variations of the drag, lift and side force coefficients with the yaw angle. (**a**) Vigorous swimming of hatchlings. (**b**) Active ascending swimming of adults.
