# Supplementary material for: Hydrodynamic role of longitudinal dorsal ridges in a leatherback turtle swimming
Source: Sci Rep. 2016 Oct 3;6:34283. doi: 10.1038/srep34283 (PMC5046118; doi:10.1038/srep34283)
Supplement: Supplementary Information [file srep34283-s1.pdf]

Hydrodynamic role of longitudinal dorsal ridges in a leatherback turtle swimming

Kyeongtae Bang[1], Jooha Kim[2], Sang-Im Lee[3, 4] & Haecheon Choi[1,3*]

Affiliations

[1]Department of Mechanical & Aerospace Engineering, Seoul National University, Seoul, Korea.

[2]School of Mechanical and Nuclear Engineering, Ulsan National Institute of Science and Technology, Ulsan, Korea.

[3]Institute of Advanced Machines and Design, Seoul National University, Seoul, Korea.

[4]Laboratory of Behavioral Ecology and Evolution, School of Biological Sciences, Seoul National University, Seoul, Korea.

[*]Correspondence to: choi@snu.ac.kr

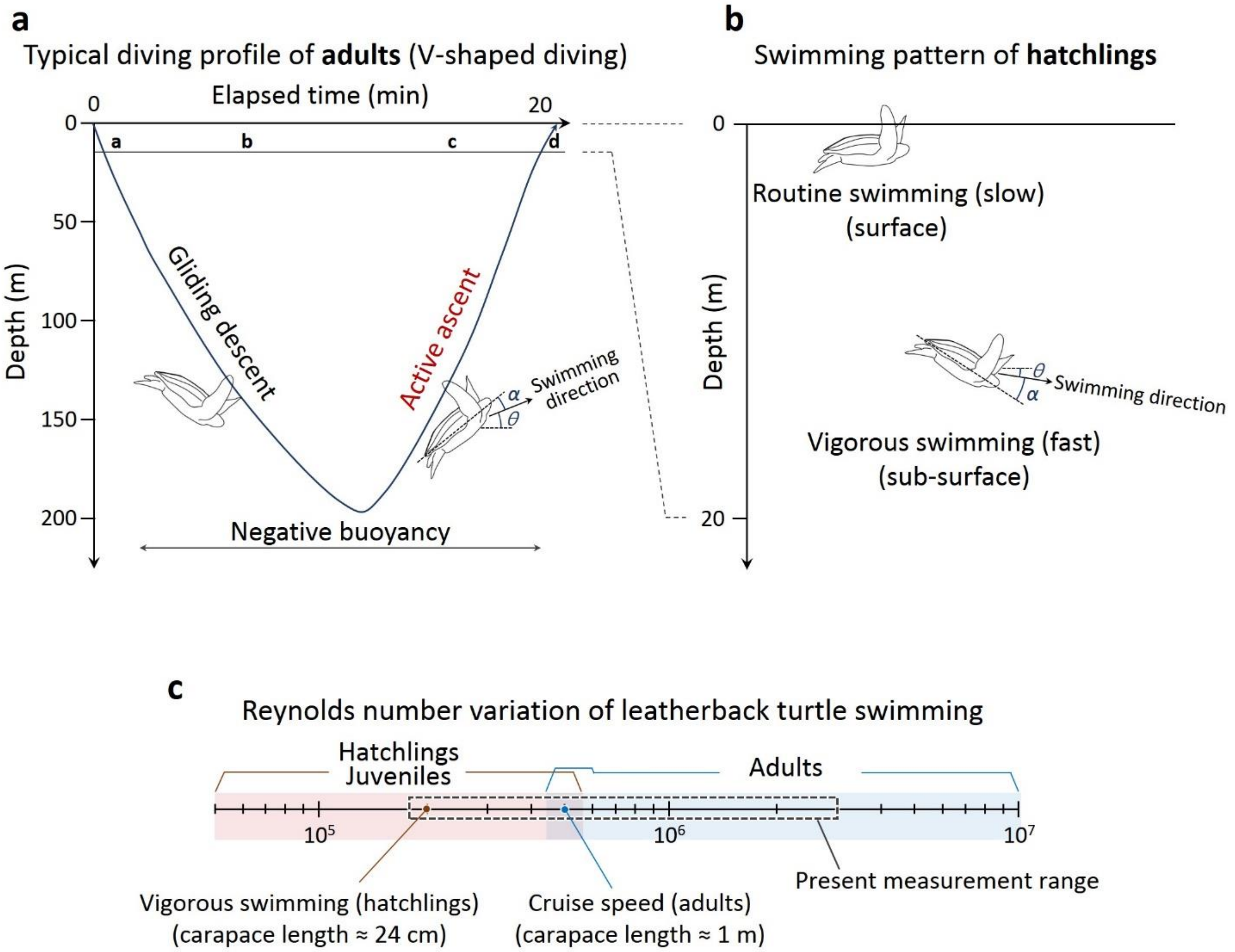

Supplementary Figure S1 | Schematic diagrams of diving patterns and Reynolds number variation of leatherback turtles. (a) Typical diving pattern of adult leatherback turtles[5]. **(b)** Swimming pattern of hatchling leatherback turtles[26]. **(c)** Variation of the Reynolds number in leatherback turtle swimming based on the carapace length and different stages of growth. Carapace length of hatchlings is about 24 cm at $Re = 2 \times 10^5$ [26] and carapace length of adults is about 1 m at $Re = 5 \times 10^5$ [21]. The present measurement range is drawn in dashed gray line. In (a) and (b), α is the angle of attack (i.e., the angle between the swimming direction and the body alignment), and θ is the angle between the swimming and horizontal directions. The values of θ are 20° - 30° during active ascending swimming[5], and near 0° during vigorous swimming[26].

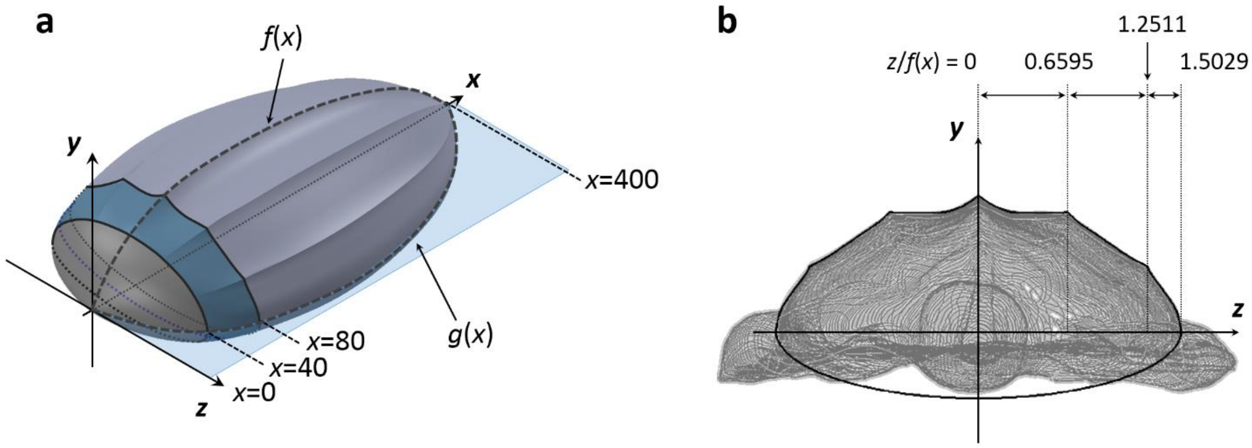

Supplementary Figure S2 | Construction of the carapace model. (a) Perspective view. **(b)** Cross-sectional view.

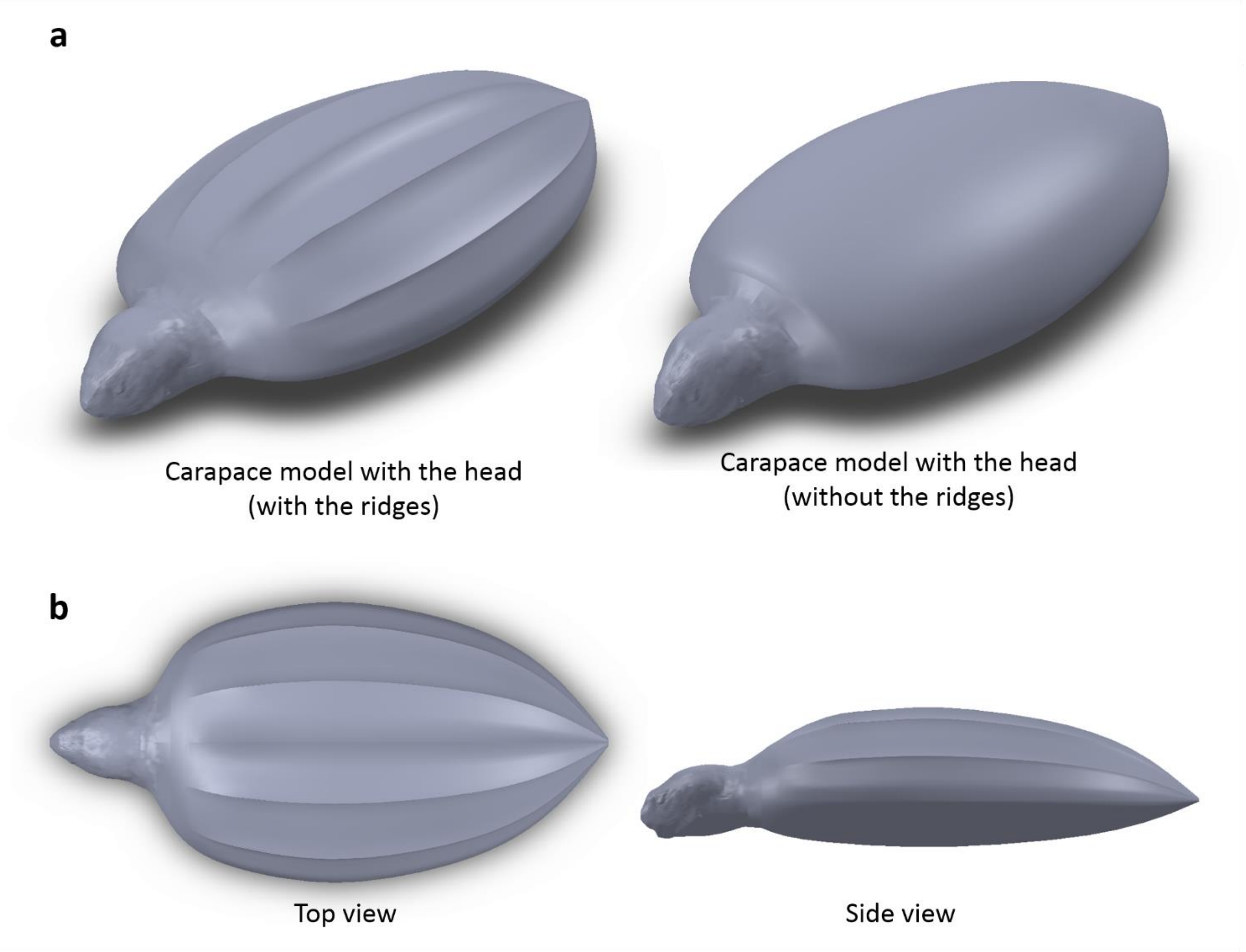

Supplementary Figure S3 | Carapace models with the head. (a) Perspective view. **(b)** Top and side views. The shape of the head was constructed based on the three-dimensional surface data of a stuffed leatherback turtle.

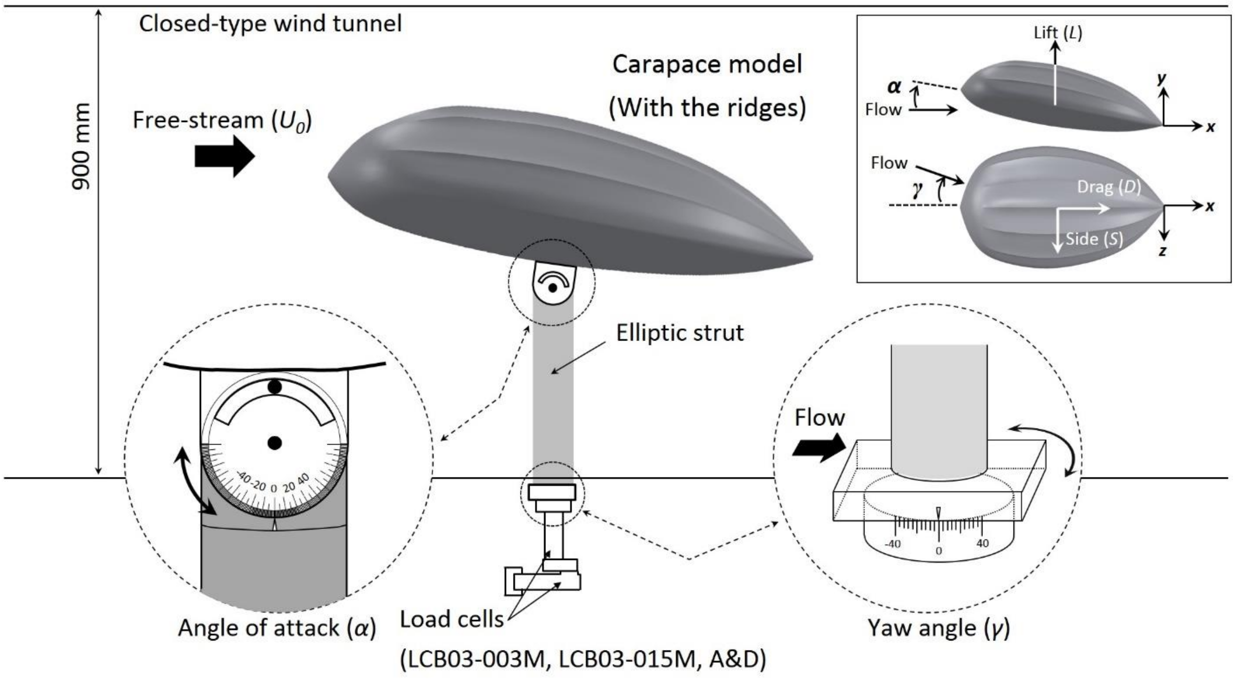

Supplementary Figure S4 | Schematic diagram of force measurements. Here, *L*, *D*, and *S* denote the lift, drag, and side forces, respectively, and γ is the yaw angle.

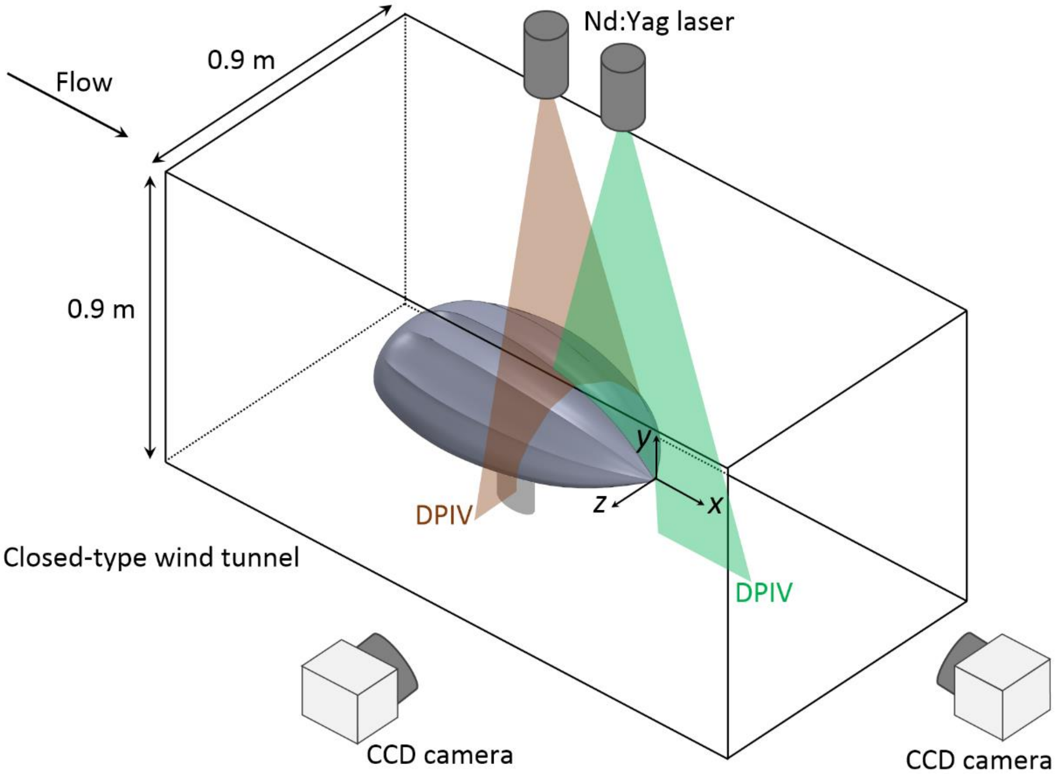

Supplementary Figure S5 | Schematic diagram of velocity measurements with DPIV.

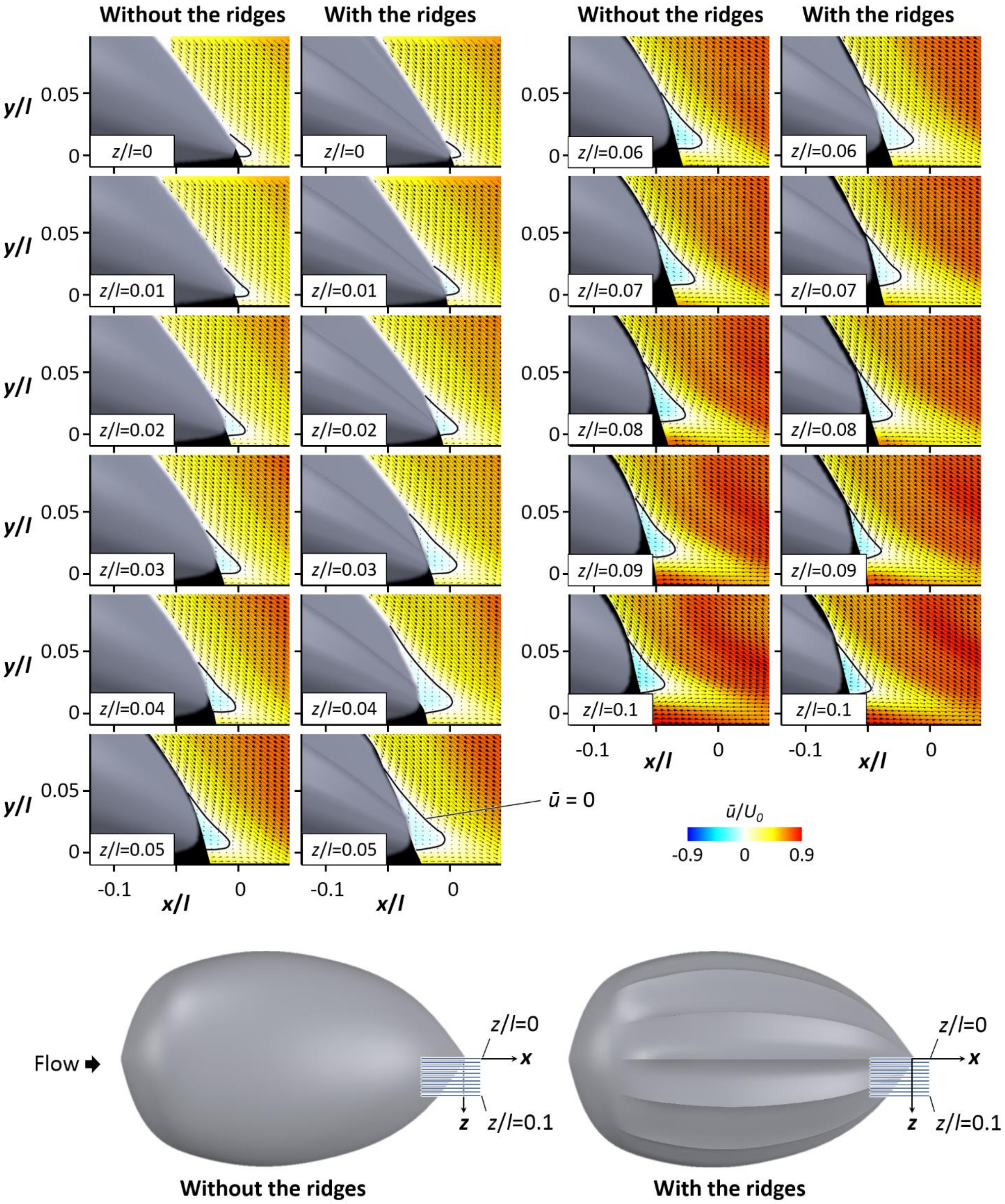

Supplementary Figure S6 | Results from velocity measurements at α = 18° and $Re = 5 \times 10^5$ that represents the active ascending swimming of adults (γ = 0º). Contours of the time-averaged streamwise velocity and velocity vectors on *x*-*y* planes at eleven spanwise locations from z/l = 0 to 0.1 with increments of 0.01. Solid black lines denote the locations where the time-averaged streamwise velocity is zero.

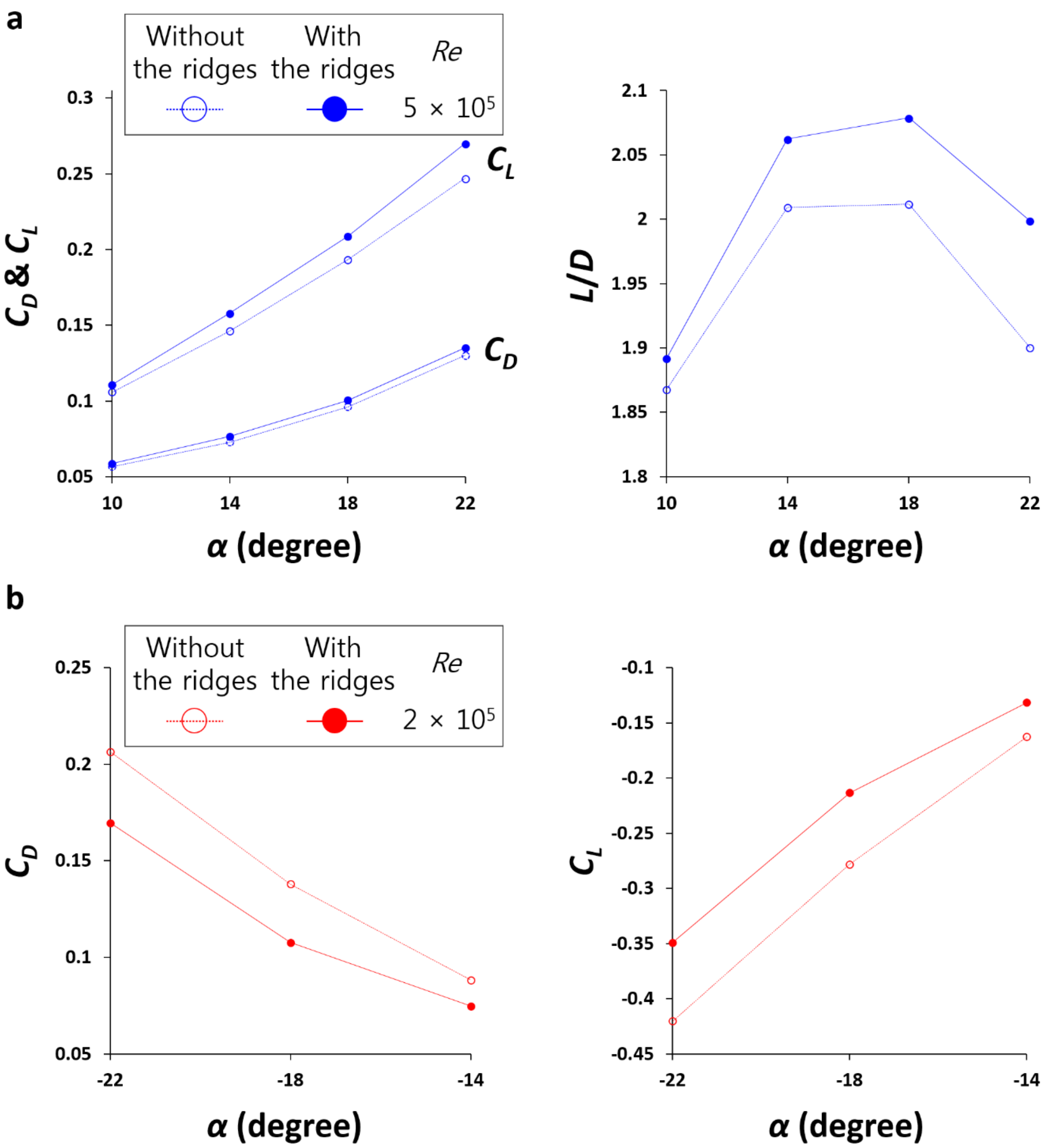

Supplementary Figure S7 | Force measurements on the carapace models with the head ($\gamma = 0^{o}$). **(a)** Active ascending swimming of adults. **(b)** Vigorous swimming of hatchlings.
